# Abstracts from German Journals

**Published:** 1872-11

**Authors:** Ch. Rauschenberg

**Affiliations:** Atlanta, Georgia


### ABSTRACTS FROM GERMAN JOURNALS.

BY CH. RAUSCHENBERG, M.D., ATLANTA, GEORGIA.

*ADDITIONS TO THE PATHOLOGY AND TREATMENT OF DIABETES MELLITUS. By Prof. Dr. O. SCHULTZEN, at Torpat, Russia.*

Physiologists do not yet fully agree as to what use is made in the human organization of the sugar which, by the process of digestion, is prepared from the amylaceous food. Some advocate the view that it is immediately consumed by combustion; others, particularly Pavy, that it is converted into fat, which is subsequently consumed in that manner.

Schermetzewsky's experiments, measuring the amount of carbonic acid produced by the human organism within a definite period of time after injections of various substances—sugar as one of them—show conclusively that the quantity of that gas is not increased by the injection of sugar, but immediately by that of the acetates and lactates of different salts and of glycerin. The exactness of the method used in these experiments makes these facts quite worthy of notice.

Prof. Dr. O. Schultzen holds that not one particle of the sugar furnished by the process of digestion is burned directly in the system, and is only partially and very indirectly connected with the production of fat. He discovered in 1866 (*Zeitschrift für Chemie*, von Hübner and Beilstern), in the urine of individuals under the influence of poisoning by phosphore, an organic substance, which formed, with zinc, a salt whose elementary composition and quantity of crystal water was strictly identical with the lactate of zinc; and later (*Charité Annalen*, v. 15), he accomplished the production of this same substance from the urine of patients who had acute atrophy of the liver; and the zinc salt which he again produced, as well as the one spoken of above, were chemically identical with the lactate of zinc, only with the difference that their solubility in alcohol was much smaller than that of the lactate of zinc, whose lactic acid has been produced from the muscular fibre of animals.

This substance is, as a late discovery has proven, the third isomeric one to lactic acid. It has been called adelhyd of glycerin, and is fully identical with the substance produced from the phosphorurine.

If we now take into consideration that the principal effect of the phosphore on the blood consists in depriving it of its faculty to combine with oxygen, while the process of digestion is not materially interfered with, and continues undisturbed, it would, if sugar is consumed directly by oxygenation, be quite logical to expect an ample supply of it in the urine of individuals poisoned by phosphore after an ample supply of sugar has been introduced by the use of amylaceous food. Chemical examination of the urine, however, in a number of such cases, did not show a particle of it in the urine. This would at once create a doubt that the sugar in the human organism is used up by oxygenation or combustion, as that process is almost entirely interrupted by phosphoric intoxication. When Dr. Schultzen, however, discovered that the quantity of glycerin-adelhyd in the urine of his cases of phosphore poisoning exhibited in quantity a direct proportion to the quantity of the amylaceous food consumed the conclusion was near on hand that this substance was the direct product of the molecular division of the sugar, but was eliminated by the kidneys on account of the deficient oxydation of the blood. This supposition became a certainty by the results of the following experiments on diabetic patients, made at Professor Schultzen's clinic.

It is perfectly plain that the faculty of the blood to combine with oxygen is not in the slightest degree diminished in diabetic patients, as all the albumen and all the substances outside of the amylaceous parts of the food are perfectly consumed by oxydation. A diabetic individual excretes, frequently, as much as one hundred grammes of urea, and more, per day; the compounds of alkalies with vegetable acids (alkaloids) are completely converted into compounds of alkalies with carbonic acid—a proof that the oxygenation is without a fault. Therefore, the sugar is excreted, unchanged, by the diabetic patient, because the ferment is wanting in his system by which the sugar, in health, is divided into glycerin and adelhyd of glycerin, according to the following equation:

$$\underbrace{H_2 \times C_6 H_{12} O_6}_{\text{Sugar.}} = \underbrace{C_3 H_6 O_3}_{\text{Glycerin-adelhyd.}} + \underbrace{C_3 H_8 O_3}_{\text{Glycerin.}}$$

Experiments on diabetic patients demonstrate that, if outside of a mere animal diet, a sufficient quantity of glycerin is taken, this latter substance is completely burned up into carbonic acid and water; that the sugar disappears from the urine almost entirely as it does when pure animal diet is taken alone—while, with the simultaneous use of glycerin, the nutrition of the most emaciated individuals improves in an astonishing manner, the sensation of weakness vanishes, the thirst abates, and all the phenomena of diabetes disappear while the patient continues this course of treatment. Whether a permanent cure can thus be accomplished, or whether the aid is only temporary, has not been decided, as these experiments have only occupied a comparatively short time. It is evident, however, that without the simultaneous use of glycerin, the diabetic patients remain feeble and emaciated, even if the excretion of sugar disappears materially. These facts explain the phenomena of diabetes in a very simple manner.

The principal materials of the body for combustion are the above products of the molecular division of the sugar and the fats. In the diabetic patient, the ferment which establishes the division of the sugar is deficient; hence, the system eliminates it undecomposed and unused. Thus the body loses a large portion of its principal material of combustion, and is burdened, besides, with the duty of carrying and eliminating this useless material. Large quantities of combustible albuminates are thus consumed; hence, the insatiable appetite—the concentration (inspissation) of the fluids of the body—the tormenting thirst, and the subsequent derangements of nutrition (cataract, tuberculosis, furunculosis.) By the use of the glycerin, the natural material for combustion, and the abstinence from amylaceous food, these disturbances are completely abated.

These facts coincide remarkably with Voit's physiological law, that the consumption of nitrogenous material in the organisms is not increased by motion and exertion of muscular power, while the production of carbonic acid, the combustion of non-nitrogenous material, invariably is.

These aphoristic data are given to induce practitioners to continue similar experiments on diabetic patients, in order to obtain a sufficient number of practical observations to confirm the theoretical basis upon which they are instituted.

The most suitable form for the administration of glycerin seems to be the following:

℞—Glycerini puriss., 20.0–50.0 (308½ ts 772 grs.)
Aquæ font., Oji.
Acid citrici seu tartar, 5.0 (77 grs.) M.
Sig.—To be taken in the course of the day.

If 60.0 (926 grs.) of glyceryin are taken, diarrhœa and nausea sometimes occur, while in the doses indicated above, it can be taken for months without any unpleasant results.—*Berliner Klin. Wochenschrift, No.* 35, 1872.

---

*ADDITIONS TO THE TREATMENT OF CHRONIC RHEUMATISM.*
*By Dr. CARL HEYMAN, Physician at Wiesbaden, Germany.*

Although the thermal waters of Wiesbaden, Teplitz, Warmbrunn, and other places of Germany—which have been used by our author, with very satisfactory results, in the most severe cases of chronic rheumatism—are only accessible to few American patients, his decidedly rational views on the pathology and treatment of this disease deserve general consideration, and, if possible, application, in the use of the thermal waters of this continent in the treatment of such cases.

Dr. Heyman describes the organic mechanism by which rheumatic disease is produced in this manner: A low degree of temperature of a moist or dry atmosphere acts as an irritant on the extremities of the sensitive nerves of the skin. If this irritation is transferred to the centers of the nervous system, and from there to neighboring or distant (particularly to predisposed) nerves, different forms of rheumatism may develop themselves: rheumatic neuralgia, when transferred to sensitive nerves; rheumatic paralysis, or spasms, when transferred to motor nerves. If transferred to vaso-motor nerves, the tissues presided over by them may, by reflex paralysis of its vessels, become more or less hyperæmic; and if the irritation of the sensitive nerves in the central organs is transferred to *near or distant trophic nerves, the tissues or organs presided over by them can take on a state of morbid inflammatory nutrition.*

Dr. Heyman thus looks upon rheumatism as a reflex phenomenon. A wet foot can, to use his own language, alike produce coryza, angina, a stiff neck, or an inflammation of the wrist, or of the ankle-joint, or other affections of that nature. If within the nerve-centers, certain provinces are peculiarly predisposed to receive impressions by the previous existence of disease within the scope of their innervation—for instance, rheumatism itself—the irritation of distant sensitive nerves, in its oscillations through the spinal marrow, can be transmitted to these predisposed portions, and can rheumatically affect the organs presided over by them. But a mere reflex paralysis of vaso-motor nerves, a change in the volume of the capillaries alone, can not produce an inflammation, according to the present standard of medical knowledge. Hence, the supposition of the existence of trophic nerves, as regulators of nutrition and metamorphosis of tissue, and as mediators of the above-mentioned inflammatory disturbances of nutrition, becomes necessary for the interpretation of this morbid phenomenon, as well as many others; and it becomes so much more admissible, as Pflüger and Eberth have demonstrated that the extremities of nerves enter the cells of glands and the cells of connective tissue, and as Meissner and others have proven beyond a doubt that certain nerve-centers preside over the nutrition of certain organs, and that irritation of the former will produce inflammatory disturbances of the latter.

If this view is correct, Dr. Heyman reasons that every coryza brought on by exposure or taking cold (so-called), every angina, every gastric catarrh or morbus Brightii, or in general every other morbid condition which is produced by the same cause, and in the same above-described manner, should be counted amongst *the rheumatoses*; or, in other words, that every tissue whose normal nutritive phenomena have, after an irritation of the nerves of the skin by extreme changes in the atmospheric temperature in the way of reflex action, been changed into an acute or chronic inflammatory disturbance, should be considered rheumatically diseased.

Fibrous tissue possesses a strong tendency, but not an exclusive privilege, to these affections. Chronic rheumatism thus represents a chronic inflammatory disturbance of nutrition, with increased metamorphosis, an increased production of new and consumption of old elements, with a fluxionary hyperæmia furnishing abundant inflammatory material. Besides this, there exists somewhere in the centers of the nervous system a state of abnormal irritability, by which the frequent changes of the disease from one locality to the other, and the so-called metastases of it, are occasioned.

The broadest therapeutical indications for the treatment of this disease are, therefore—

1. To promote a state of the system which diminishes secretion and increases resorption, and thereby establishes restitution of the affected tissues.

2. To induce a normal and uniform degree of excitability of the nerve-centers.

In relation to the first indication, the hot springs, the so-called thermal waters, present the most effective remedy, as every experienced physician certainly resorts to the customary medication of such cases only from old habit, or in self-defense, in order to avoid the reproach of indifference and carelessness, but certainly always with reluctance, and with little or no hope of improving the condition of his patient materially.

Dr. Heyman reasons that *the principal effect of warm baths, of the temperature of the blood, or warmer, consists in a physical dilatation of the capillaries of the surface,* which continues some time after the bath, and can be maintained almost any length of time by the warmth of the bed to which the patient must be confined more or less permanently. O. Weber and Falk* show that this dilatation of the capillaries does not materially depend upon vaso-motor nerve influence, and does take place, even after the vascular nerves have been divided, by the physical effect of the heat on the textures of the tissues of the capillaries. This fact has also been demonstrated by Dr. Heyman on a patient with complete left-sided hemiplegia, and subsequent anæsthesia, on whom, by the influence of hot-water baths, both hands became equally red and turgid—on the sound side in five minutes, on the paralyzed one in fifteen to twenty minutes, with the peculiarity that the disappearanee of that redness and turgidity on the diseased side occupied a much longer space of time than on the well one.

This relaxation of the organic fibre by physical influence does

* Virchow's Archives, Vol. liii, No. 1. Atlanta Medical and Surgical Journal, August, 1872, page 291.

not only produce dilatation of the capillaries, but also an expansion of the surrounding tissues, permitting the pressure of the blood to expand the capillaries still more. *A cutaneous hyperœmia of the surface, coupled with a relatively corresponding anœmia of the interior tissues*, is, therefore, the primary and principal effect of hot baths, which causes (1) a diminution of the morbid afflux of blood to the diseased tissues, and therefore a diminution of exudates and deposits, and (2) increased resorption in consequence of diminished quantity of blood in the capillaries of the diseased tissues.

While warm bathing thus dilates the capillary diameters, it also diminishes the resistance of the walls of these vessels to the pressure of the heart, and increases the velocity of the blood-current; while, at the same time, by this diminished resistance, the normal mechanical irritation of the heart, through the force of the backward pressure of the blood-wave on the heart, is also diminished, and therefore the frequency of the contractions of that organ decreased. Actual experiments prove that the pulse of different individuals in the warm bath increases or decreases as the one or the other momentum affects them more prominently.

Other effects of warm bathing—for instance, the increased temperature of the body, and the possibility of too great excitation of the nervous system—although not so important, must be remembered and brought to bear on such cases, or must be avoided, according to circumstances.

These collateral effects depend principally upon the chemical character of the water used. In relation to this item, the waters of hot springs are divided into those which contain only indifferent chemical substances, (simple thermal waters;) those which contain saline substances, principally chloride of sodium, and those which contain principally sulphur.

All those thermal waters whose only or far most effective dynamic element is their high temperature, must be counted amongst the simple thermal waters, and hence those that do not contain gaseous or saline substances in sufficient quantities to produce, by their effect upon the sensitive nerves of the surface a marked irritation of the nervous system must be brought under this class; for instance, the hot waters of Teplitz, Warmbrunn, Leuk and Wiesbaden, and they are principally used in these cases.

Those hot springs which contain a large amount of gas (carbonic acid) cause not only a direct relaxation of the cutaneous vessels, but increase that effect indirectly by reflex paralysis of the cutaneous vaso motor nerves.

The same applies to the hot springs containing sulphur. Dr. Heyman has demonstrated experimentally that sulphuretted hydrogen gas brought in contact with the skin has a highly exciting effect upon the nervous system.

He considers the localities where the simple thermal waters can be used the most suitable resorts for rheumatic patients, as they favor a restitution of the morbid tissues without too great an irritation of the nerve centers, and can, therefore, be used more methodically, longer at a time and oftener in succession.

The temperature and duration of the single bath must be accommodated to the individuality of each patient and the irritability of his nervous system. As a general rule it may be said that a patient generally need not remain longer than one hour in the bath, or use water warmer than 30° C. (68° F.) If the trouble is of a remittent character, the cutaneous hyperæmia induced by the bath must be carefully kept up by the warmth of the bed. Such patients must, during the commencement of the treatment, remain in bed permanently, and from five to six hours after each bath, even if an improvement becomes plainly perceptible. Bathing in the morning, with one hour's rest in bed, followed by unrestricted exposure to the changes of the atmosphere the balance of the day is poor treatment in the best of cases. Painful sensations of the affected parts, or pressure, on motion, indicate the necessity of the greatest possible amount of rest and comfortable posture, favoring the return of the blood from those parts. Cautious and gradual, passive and active motions, are indicated as soon as these painful sensations disappear. Light and careful exercise, not carried on to fatigue, on sunny, warm and dry days, may be allowed after the motory organs have become free of all pain. Occasionally warm douches, warm cataplasms or similar cutaneous irritations can be resorted to, to promote the resorption of indolent local products of the chronic inflammation. Everything tending to promote resorption and a profuse action of all the secretory and excretory organs must be regulated by the simultaneous use of proper mineral waters or otherwise. The diet should be scrupulously controlled; all irritating articles of food, and all excesses in eating, should be carefully avoided. Milk diet, therefore, occupies the first and highest rank as an additional item of success in the treatment of these cases.

As the patient is compelled to remain in bed a large portion of his time, he should occupy a roomy, well-ventilated apartment, fronting to the south, if possible.

After the completion of the cure, Dr. Heyman uses, according to the individuality of his patients, saline or sea-baths, or a rational hydropathic treatment, or an iron water containing carbonic acid, in order to restore the tone of the relaxed skin and its adaptability to the changes of the atmospheric temperature.

He admits that heated spring or river water, used at home, would answer as good a purpose, if, during the entire duration of the treatment, all hostile influences could be kept from irritating the cutaneous extremities or the centers of the nervous system. The difficulty of a methodical treatment at home, the uniformly temperate climate, the required lower or higher altitude, the bland and simple diet to which the patient will submit much better under unusual surroundings than at home, and the alterative effect upon the physical and mental condition of the patient, resulting from the change and transformation of his surroundings and mode of life make the treatment of these cases, at a pleasant watering place, where all these conditions exist, generally much more successful than at home.

Dr. Heyman speaks of the above described method of treatment of chronic rheumatism, from his experience at Wiesbaden, as decidedly successful and satisfactory, even in very stubborn cases.

In discussing the contra-indications to this method of treatment by hot baths, he again calls attention to the fact that *increased tone of larger provinces of blood-vessels causes, by the increased resistance to the blood-wave, more energetic action of the heart, decreased tone of larger provinces of blood-vessels, decreased action of the heart.**

*Vide Goltz's views; ATLANTA MEDICAL AND SURGICAL JOURNAL, August, 1872, page 293.

This circumstance must be well remembered, when elderly individuals, with loose flabby muscles and perhaps a diseased heart, require treatment. The hot baths in such cases are not only of very doubtful propriety, but often really dangerous, as by the dimunition of the vascular tone over the entire surface of the body, they cause a relaxation of the heart, which has occasionally been followed by sudden death. Thus, wherever a suspicion of fatty degeneration of the heart or of an atheromatous condition of the coronary arteries, or symptoms of congestion of the brain, with cold extremities, sensation of weakness and fainting, a weak or intermittent pulse, atheromatous hardness of the arteries that can be felt, do exist, this method of treatment is inadmissible.

The sensations of faintness after too sudden a removal of watery exudations, for instance, after puncture in ascites, the weakening effect which the warmth of the bed has on the action of the heart in aged individuals depend probably upon the same cause.

Diseases of the brain, predisposition to congestion of the brain, diseases of the spinal marrow, particularly tabes dorsalis, furnish, as a matter of course, further contra-indications to this method of treatment.—(*Berliner Klinische Worshenschrift, No*. 37, 1872.)

